# Editorial: Molecular and cellular pathways leading to mitochondrial dysfunction and neurodegeneration: lessons from *in vivo* models, volume II

**DOI:** 10.3389/fnins.2025.1557013

**Published:** 2025-01-29

**Authors:** Shabab B. Hannan, Federica De Lazzari, Aurora Gomez-Duran, Juan A. Navarro, Gloria Brea-Calvo, Alvaro Sanchez-Martinez

**Affiliations:** ^1^Jan and Dan Duncan Neurological Research Institute and Texas Children's Hospital, Houston, TX, United States; ^2^Department of Neurology, Baylor College of Medicine, Houston, TX, United States; ^3^MRC Mitochondrial Biology Unit, University of Cambridge, Cambridge, United Kingdom; ^4^MitoPhenomics Lab, Biological Research Center Margarita Salas-CSIC, Madrid, Spain; ^5^INCLIVA Biomedical Research Institute, Unversitat de València, Valencia, Spain; ^6^Andalusian Center for Developmental Biology (CABD), CSIC/Universidad Pablo de Olavide/Junta de Andalucía, and CIBERER (ISCIII), Seville, Spain

**Keywords:** mitochondria, *Drosophila*, Parkinson's disease, *ATAD3*, SARS-CoV-2, Usp14, metformin, *Pink1*

The second volume of our editorial is a continuation of the first (Hannan et al., 2022) and features two reviews and three research articles that highlight how mitochondrial dysfunction can cause neurological diseases and suggest potential therapeutic targets for Parkinson's disease (PD) and neurological effects of SARS-CoV-2 infection. The research discussed in this special edition highlights the multifaceted nature of mitochondrial biology, with reviews on mitochondrial protein synthesis, *ATAD3* mutations and their developmental impact, as well as research on sleep dysregulation, PD, and the neurological effects of SARS-CoV-2.

Brügel et al. provide a comprehensive review of studies investigating the role of mutations in *ATAD3*, which encodes a mitochondrial membrane protein associated with the inner mitochondrial membrane structure, nucleoid organization, cholesterol trafficking, and lipid metabolism, but whose primary function is unclear. Mutations in *ATAD3* result in several human diseases, including developmental delay, optic atrophy, muscular atrophy, peripheral neuropathy, and congenital cataracts. This review brings together findings from *Drosophila* and mice model studies exploring the effects of several pathogenic mutations, including muscle- and neuron-specific ablation of *ATAD3* function. In mice, ATAD3 dysfunction leads to progressive myopathy and premature death, highlighting the central role of this protein in cellular survival. In *Drosophila*, some variants are associated with embryonic lethality and increased mitophagy, while others show partial or failed lethality rescue in loss-of-function models. These variants caused locomotion defects, small mitochondria with cristae abnormalities, accumulation of autophagic intermediates, and cholesterol aggregates. The authors offer an in-depth overview of the current state of *ATAD3* research, providing a useful resource for investigators interested in its function.

For the second review article in this special research edition, Antolinez-Fernández et al. explore the critical role of mitochondrial protein synthesis in neurodegenerative and muscular diseases. Despite its bacterial origin, mitochondrial protein synthesis is a highly specialized process that differs significantly from prokaryotic translation. Impairment in this process has been implicated in a wide array of diseases. The authors provide a detailed molecular overview of mitoribosome structure and different stages of mitochondrial protein translation. Although only 13 of the ~1,100 mitochondrial proteins are encoded by the mtDNA, several diseases are directly linked to mutations in mitochondrial protein synthesis. The review emphasizes the importance of understanding the molecular mechanisms underlying mitochondrial protein translation to develop therapeutic strategies for disorders caused by defects in this process, which are multisystemic and often associated with neurological symptoms. Future advancements in gene editing techniques may offer deeper insights into the tissue-specific impact of mitochondrial translation dysfunction that will probably help to understand the clinically heterogeneous consequence of defects in this process.

A research article by Favaro et al. explores the potential therapeutic benefits of downregulating Usp14 function, a deubiquitinating enzyme, in a *Pink1* knockout (KO) fly model of PD. PD is a progressive neurodegenerative disorder primarily characterized by the loss of dopaminergic neurons in the brain, leading to motor dysfunction and other neuropsychiatric symptoms. One of the hallmark features of PD is mitochondrial dysfunction, which plays a critical role in the progression of the disease. Favaro et al. demonstrated that downregulation of *Usp14* in *Pink1* KO flies rescued both circadian rhythms and sleep disturbances. While the mechanisms behind the improved functions remain unclear, Usp14 inhibition may function by promoting proteostasis and reducing the buildup of dysfunctional proteins and mitochondria that contribute to neurodegeneration. This study suggests that targeting *Usp14* could provide a novel therapeutic approach for early intervention in PD, potentially improving circadian rhythm disturbances and sleep defects before the onset of motor symptoms. Moreover, the availability of Usp14 inhibitors that are effective in mice and can cross the blood-brain barrier makes this a promising avenue for PD treatment.

Ay et al. found that Mito-Met, a metformin analog, exerts neuroprotective effects in cell and mouse models of PD by activating PKD1, Akt, and AMPK pathways. Mito-Met protects against neurotoxicity, mitochondrial fragmentation, and dopamine depletion, indicating its potential as a therapeutic agent for PD that warrants further evaluation.

Lastly, Guo et al. examine the effects of SARS-CoV-2 infection in neuronal vulnerability. The study focuses on Nsp7, a non-structural protein encoded by the viral genome. Both *in vivo* and *in vitro* studies suggest that expression of Nsp7 results in reduced levels of synaptic proteins. Furthermore, expression of Nsp7 leads to oxidative stress and impaired ATP production. Interestingly, treatment with antioxidant N-acetylcysteine *in vitro* rescued the deleterious effect of Nsp7 expression on synaptic proteins PSD95 and synaptophysin. This research points to the role of mitochondria in COVID-19-related neurological disorders, offering a potential target for therapeutic intervention. The findings suggest that mitochondrial damage may be an important factor in the neurological manifestations of COVID-19, including memory deficits and other cognitive impairments.

**Figure 1:**
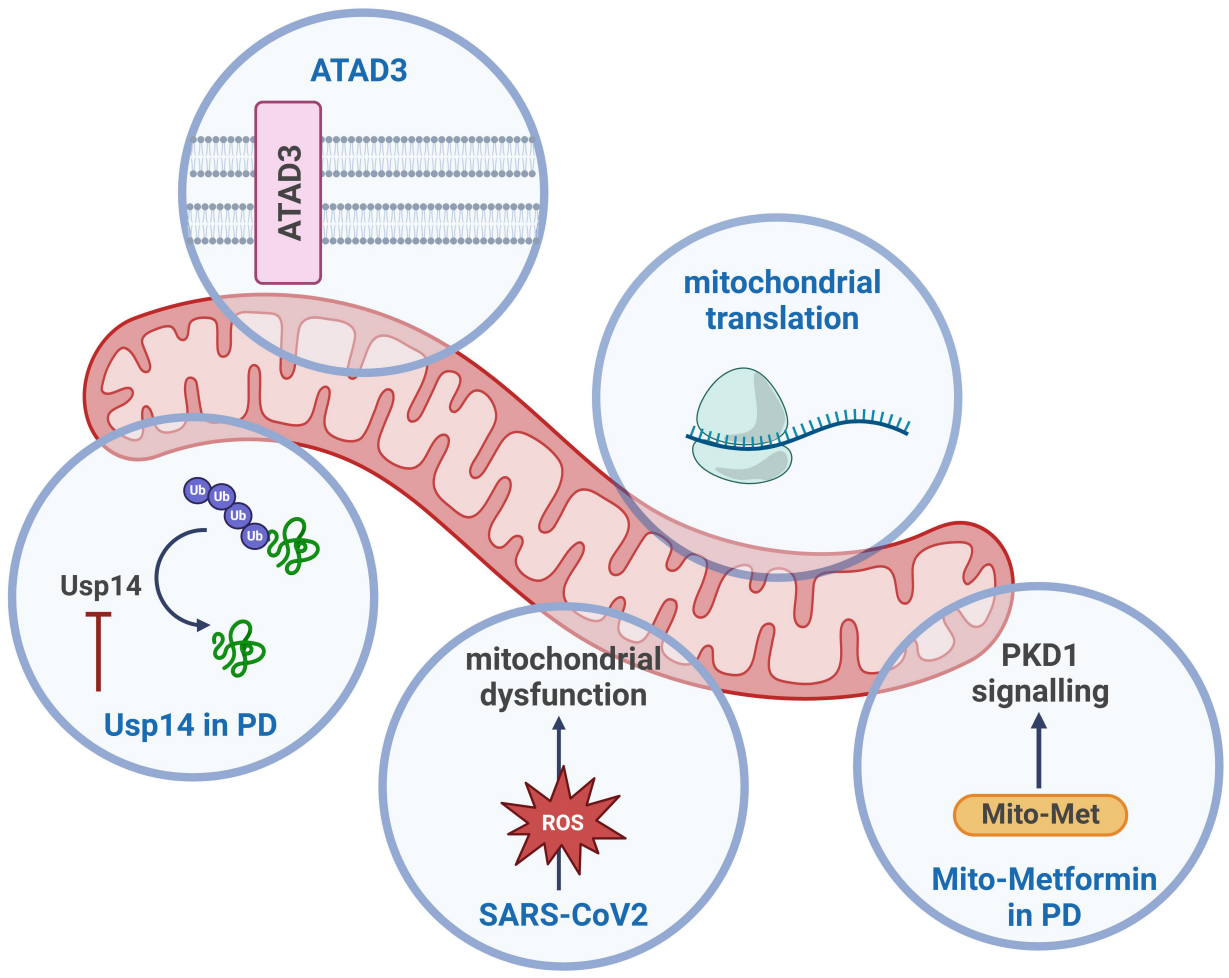
Schematic overview of the mitochondrial processes discussed in this Research Topic. Created with BioRender.com.

In summary, these articles collectively underscore the diverse and critical roles of mitochondria in various neurological diseases, from neurodegenerative and neurodevelopmental disorders to the neurological effects of viral infections like SARS-CoV-2 (see Figure 1). The research underscores the importance of mitochondrial function in cellular health and highlights the promising potential of targeting mitochondrial pathways for disease treatment.
